# The Bay Area Verbal Learning Test (BAVLT): Normative Data and the Effects of Repeated Testing, Simulated Malingering, and Traumatic Brain Injury

**DOI:** 10.3389/fnhum.2016.00654

**Published:** 2017-01-12

**Authors:** David L. Woods, John M. Wyma, Timothy J. Herron, E. William Yund

**Affiliations:** ^1^Human Cognitive Neurophysiology Laboratory, Veterans Affairs Northern California Health Care SystemMartinez, CA, USA; ^2^University of California Davis Department of NeurologySacramento, CA, USA; ^3^Center for Neurosciences, University of California DavisDavis, CA, USA; ^4^University of California Davis Center for Mind and BrainDavis, CA, USA; ^5^NeuroBehavioral Systems, Inc.Berkeley, CA, USA

**Keywords:** aging, reaction time, memory, dementia, education, sex, head injury, malingering

## Abstract

Verbal learning tests (VLTs) are widely used to evaluate memory deficits in neuropsychiatric and developmental disorders. However, their validity has been called into question by studies showing significant differences in VLT scores obtained by different examiners. Here we describe the computerized Bay Area Verbal Learning Test (BAVLT), which minimizes inter-examiner differences by incorporating digital list presentation and automated scoring. In the 10-min BAVLT, a 12-word list is presented on three acquisition trials, followed by a distractor list, immediate recall of the first list, and, after a 30-min delay, delayed recall and recognition. In Experiment 1, we analyzed the performance of 195 participants ranging in age from 18 to 82 years. Acquisition trials showed strong primacy and recency effects, with scores improving over repetitions, particularly for mid-list words. Inter-word intervals (IWIs) increased with successive words recalled. Omnibus scores (summed over all trials except recognition) were influenced by age, education, and sex (women outperformed men). In Experiment 2, we examined BAVLT test-retest reliability in 29 participants tested with different word lists at weekly intervals. High intraclass correlation coefficients were seen for omnibus and acquisition scores, IWIs, and a categorization index reflecting semantic reorganization. Experiment 3 examined the performance of Experiment 2 participants when feigning symptoms of traumatic brain injury. Although 37% of simulated malingerers showed abnormal (*p* < 0.05) omnibus z-scores, z-score cutoffs were ineffective in discriminating abnormal malingerers from control participants with abnormal scores. In contrast, four malingering indices (recognition scores, primacy/recency effects, learning rate across acquisition trials, and IWIs) discriminated the two groups with 80% sensitivity and 80% specificity. Experiment 4 examined the performance of a small group of patients with mild or severe TBI. Overall, both patient groups performed within the normal range, although significant performance deficits were seen in some patients. The BAVLT improves the speed and replicability of verbal learning assessments while providing comprehensive measures of retrieval timing, semantic organization, and primacy/recency effects that clarify the nature of performance.

## Introduction

Binet and Henri (1894) first developed verbal learning tests (VLTs) to evaluate the memory of Parisian schoolboys. Influenced by Binet's work, the Swiss neurologist Claparède adapted VLTs for use in the clinical examination of patients with memory disorders (Boake, 2000), and Claparède's student, André Rey, systematized the tests in the Rey Auditory Verbal Learning Test (RAVLT) (Rey, 1958). The RAVLT was translated into English (Rey and Benton, 1991) and has since undergone multiple revisions.

Verbal learning tests are widely used to measure learning disabilities and attention-deficit hyperactivity in children (Van Den Burg and Kingma, 1999; Waber et al., 2007; Vakil et al., 2010), and to evaluate verbal memory in adults with mild cognitive impairment and dementia (Delis et al., 2010; Crane et al., 2012; Fernaeus et al., 2014; Mata et al., 2014), traumatic brain injury (TBI) (Vanderploeg et al., 2001; French et al., 2014), concussion (Collie et al., 2003; Lovell and Solomon, 2011), stroke (Baldo et al., 2002), and substance abuse (Price et al., 2011).

Table 1 summarizes the properties of current VLTs, including the RAVLT-II (Geffen et al., 1990), the California Verbal Learning Test (CVLT-II) (Delis et al., 2000), and the Hopkins Verbal Learning Test (HVLT) (Brandt, 1991). The RAVLT requires about 15 min to administer and another 5 to 10 min to score. It includes five presentations of a 15-word list (List A). This list is read at 1 s intervals between words, with each presentation followed by immediate list recall. After the fifth presentation of List A, a 15-word distractor list (List B) is presented for recall, followed by the immediate recall (IR) of List A. After a 20 min delay, the participant again recalls the words in List A in a delayed recall (DR) trial. Finally, there is a recognition test in which participants are asked to recognize the 15 words from List A among a set of 50 words which include the words from List A and List B as well as 20 words that are semantically related to the List A words.

**Table 1:** **Properties of verbal learning tests**.

	**Words**	**Reps**	**B-list**	**IR**	**DR**	**Recog**	**Semantic**	**Rate**	**Admin time**	**Score time**
RAVLT	15	5	Y	Y	Y	N	N	2 s	15	5-10
HVLT	12	3	N	N	Y	Y	Y	2.5 s	10	2-3
CVLT	16	5	Y	Y	Y	Y	Y	1 s	30	15-25
BAVLT	12	3	Y	Y	Y	Y	Y	1.93 s	10	0

*Words, number of words in each list; Reps, number of repeated presentations of list A; B-list, includes distractor list; IR, immediate free recall; DR, delayed free recall; Recog, recognition trial; Semantic, includes different semantic categories; Rate, rate of word presentation in words/s; Admin time, administration time in minutes. Score time, scoring time in minutes; RAVLT, Rey Auditory Verbal Learning Test; HVLT, Hopkins Verbal Learning Test; CVLT, California Verbal Learning Test; BAVLT, Bay Area Verbal Learning Test described in this manuscript*.

The CVLT-II (Delis et al., 2000) is the most demanding VLT, requiring up to 30 min to administer and an additional 15 to 25 min to score. It uses 16-word lists that belong to four different semantic categories. Like the RAVLT, List A is presented five times, followed by a distractor List B, and the immediate recall of List A. The CVLT then adds a semantically-cued recall trial in which participants are asked to name all of the words in each of the four semantic categories. After a 20-min delay, a delayed-recall trial is administered, followed by a delayed cued-recall trial. Finally, participants are given a yes/no recognition test with list A words mixed with list B words, words semantically and phonemically related to list A words, and unrelated novel distractors.

The HVLT (Brandt, 1991) is the shortest VLT designed for difficult-to-test subjects: It can be administered and scored in less than 15 min. The HVLT uses 12-word lists which are presented three times, followed by a recognition trial. The revised version, (HVLT-R) (Benedict et al., 1998) added a 20–25 min delayed recall trial before a yes/no recognition trial with 24 words.

Table 2 provides a summary of results of normative studies using the RAVLT, HVLT, and CVLT, including retrieval scores on List A1, scores on the final List A presentation (A5 on the RAVLT and CVLT, and A3 on the HVLT), total acquisition scores (the number of words recalled on all List A presentations), scores on the distractor list (List B), and IR and DR scores. Crossen and Wiens (1994) compared the performance of subjects on the RAVLT and the CVLT and found slightly higher scores on the CVLT, which may reflect its longer word lists (16 words vs. 15 words on the RAVLT). However, Pearson correlations between corresponding measures on the two tests were surprisingly low, ranging from *r* = 0.12 for List B to *r* = 0.47 for total acquisition scores. Somewhat higher correlations (*r* = 0.30 to 0.74) have been found between the HVLT and CVLT (Lacritz and Cullum, 1998; Lacritz et al., 2001).

**Table 2:** **Summary results from normative studies of verbal learning**.

**Study**	***N***	**Age**	**Edu**	**A1**	**A3/A5**	**Total acq**	**List B**	**IR**	**DR**
**RAVLT**									
McMinn et al., 1988	209	29.1(6.0)	14.5(1.5)	7.4(1.8)	13.1(1.8)	55.4(7.9)	6.7(1.7)	11.9(1.6)	
Geffen et al., 1990	152	44.5(20.2)	11.2(2.2)	6.6(1.6)	11.6(1.8)	48.7(7.4)	5.8(1.7)	10.0(2.2)	10.1(2.5)
Magalhães and Hamdan, 2010	302	50.6(15.9)	11.3(3.7)	6.6(2.1)	11.7(2.3)	48.1(9.6)	6.2(2.3)	9.6(2.7)	9.9(2.7)
Uchiyama et al., 1995	1818	36.9(7.2)	16.0(2.4)	6.7(1.8)	12.6(1.9)		6.5(2.1)	11.2(2.7)	10.9(2.8)
Crossen and Wiens, 1994	100	29.9(6.2)	14.7(1.6)	7.0(1.6)	12.2(1.8)	51.7(7.5)	7.0(2.0)	10.6(3.1)	
Knight et al., 2006	228	73.7(5.8)	12.5(2.3)	5.2(1.7)	11.0(2.5)	43.4(9.3)	4.7(1.7)	8.4(3.2)	8.0(3.5)
Speer et al., 2014	407	61.7(5.8)	12.3(3.9)	7.3(1.8)	13.0(1.7)	53.8(7.9)		11.0(2.6)	11.1(2.6)
Gale et al., 2016	177	75.9(8.0)	15.5(2.7)			45.8(9.0)			9.4(2.7)
**HVLT**									
Benedict et al., 1998	531	48.1(17.3)	13.8(2.3)	7.9(1.7)	10.8(1.3)	28.5(4.0)			10.3(1.8)
Vanderploeg et al., 2000	394	73.0(12.0)	14.1(2.3)	4.8(1.7)	8.4(2.2)	20.6(5.2)			7.8(2.7)
Hester et al., 2004	203	73.1(5.6)	11.1(3.1)	5.8(1.7)	9.0(2.1)	22.5(5.6)			7.6(3.2)
Norman et al., 2011	143	37.6(7.3)	14.1(2.4)			29.2(3.9)			10.4(1.9)
Duff, 2016	290	77.1(7.5)	15.4(2.6)	6.6(1.9)	9.6(2.1)	24.8(5.7)			7.4(3.6)
**CVLT**									
Norman et al., 2000	672	51.2(20.3)	13.7(2.6)	7.0(2.2)	12.7(2.6)	51.3(9.5)	6.6(2.2)	10.4(3.2)	10.7(3.3)
Norman et al., 2000	234	53.3(18.3)	13.8(2.6)	7.4(1.9)	12.2(2.6)	51.5(11.5)	6.6(2.3)	10.4(3.5)	10.8(3.6)
Crossen and Wiens, 1994	100	29.9(6.2)	14.7(1.6)	7.5(1.6)	13.0(1.8)	55.1(7.7)	7.9(1.9)	11.7(2.3)	
Wiens et al., 1994	700	29.1(6)	14.5(1.6)			56.0(7.6)			
Paolo et al., 1997	212	70.6(7.0)	14.9(2.6)	5.8(1.8)	10.8(2.3)	44.6(8.9)	5.5(2.0)	8.9(2.8)	9.3(2.7)
Woods et al., 2006	195	48.4(22.3)	14.17(2.4)	6.0(1.8)	11.5(2.9)	47.3(11.5)	5.3(2.1)	9.9(3.6)	10.3(3.6)
**BAVLT**									
Current study	195	40.3(20.2)	14.7(2.1)	5.6(1.8)	9.0(2.1)	22.5(5.3)	6.1(1.9)	7.4(2.6)	7.2(2.7)

*Edu, years of education; A1, mean number of words retrieved on first presentation of List A; A3/A5, words retrieved on the final list A presentation (List A is presented three times in the HVLT and BAVLT, and five times in the RAVLT and CVLT); Total acq, sum of words retrieved on all list A acquisition trials; List B, words retrieved on the distractor list; IR, immediate recall; DR, delayed recall; Standard deviations are shown in parentheses. Note that RAVLT presents a 15-word list five times (maximum total acquisition score = 75), the HVLT presents a 12-word list three times (maximum score = 36), the CVLT presents a 16-word list five times (maximum score = 80), and the BAVLT presents a 12-word list three times (maximum score = 36)*.

Most normative data sets report the scores for List A1, total acquisition, List B, IR, DR, and recognition. More innovative scoring measures have also been developed. For example, several groups have analyzed primacy and recency effects (Martin et al., 2013; Egli et al., 2014; Fernaeus et al., 2014), semantic clustering (Woods et al., 2005), and the type and incidence of errors (Savage and Gouvier, 1992; Lacritz et al., 2001; Woods et al., 2005). Vakil et al. (2010) developed a number of additional RAVLT measures, including learning rate (A5–A1), corrected total learning (total acquisition–5^*^list A1 scores), proactive interference (A1–B), retroactive interference (A5–IR), retention (A5–DR), and retrieval efficiency (recognition–DR).

The CVLT is the most popular verbal learning test (Rabin et al., 2016), in part because its scoring program provides an extensive set of measures, including six primary measures and 28 “process measures,” which include scores on individual trials (A1, A5, and B), clustering measures (semantic, serial, and subjective), learning slopes, across-trial consistency measures, primacy and recency effects, cued and free-recall intrusions, repetitions, and four different recognition measures including source recognition, novel recognition, semantic recognition, and recognition bias.

### Normative data consistency

Although verbal learning tests generally show good test-retest reliability within a laboratory, discrepancies in normative data collected from different laboratories have been noted in a number of studies. For example, Wiens et al. (1994) gathered norms from 700 healthy job applicants using the CVLT and found mean scores that were about 0.4 standard deviations below those obtained from the age-matched norms of the original CVLT normative sample (Delis et al., 1987). Similarly, Paolo et al. (1997) studied 212 elderly controls and found results that were significantly below the original CVLT norms for older adults. Similar variations have also been found in different RAVLT norms (McMinn et al., 1988; Geffen et al., 1990; Savage and Gouvier, 1992). As a result, when Stallings et al. (1995) analyzed the performance of patients with moderate and severe traumatic brain injury (TBI) on the RAVLT and CVLT, the percentage of patients showing abnormalities ranged from 35% to 85% depending on which test and normative data set were used for comparison.

### Examiner effects

Because VLT scores are influenced by the clarity and rate of word presentation, differences between examiners can arise due to variations in the accent, intonation, audibility, and pace of word delivery. For example, Wiens et al. (1994) compared the CVLT scores of demographically similar participant groups when tested by seven doctoral-level examiners. They found between-examiner differences of nearly one standard deviation and noted that “…Further research will be needed to evaluate possible examiner-related test administration characteristics, such as rate of word presentation and accuracy of recording as well as examiner-subject empathy and capacity to elicit best effort from subjects.” Concerns about inter-examiner differences in list presentation led Van Der Elst et al. (2005) to use computerized word delivery. However, although Van Den Burg and Kingma (1999) used recorded word lists they still found significant inter-examiner differences, although of a smaller magnitude than those reported by Wiens et al. (1994).

### The Bay Area Verbal Learning Test (BAVLT)

Here we describe the psychometric properties, reliability, and clinical application of the computerized Bay Area Verbal Learning Test (BAVLT). The BAVLT eliminates inter-examiner differences in list presentation, simplifies response recording, and provides a complete digital record of the test that includes the time of occurrence of each response. In addition, scoring is automated, reducing errors that may occur when tallying and classifying responses. Finally, the BAVLT provides a comprehensive set of automated scoring metrics, similar to those of the CVLT-II, while reducing the total time of VLT administration and scoring to approximately 10 min.

In Experiment 1, we describe the baseline performance characteristics of 195 control participants ranging in age from 18 to 82 years. In Experiment 2, we examine BAVLT test-retest reliability and the influence of learning on performance in 29 young participants who underwent three test sessions at weekly intervals. In Experiment 3, we examine the performance of Experiment 2 participants when instructed to simulate deficits due to traumatic brain injury (TBI). Finally, in Experiment 4, we describe the performance of patients with mild and severe TBI.

## Introduction: Experiment 1

In Experiment 1, we investigated the influence of demographic variables, including age, education, and sex, on BAVLT performance. Previous studies have shown that VLT performance declines markedly with advancing age (Wiens et al., 1994; Paolo et al., 1997; Benedict et al., 1998; Delis et al., 2000; Steinberg et al., 2005; Magalhães and Hamdan, 2010). We therefore anticipated significant age-related declines in the BAVLT. We also anticipated that older subjects would show a reduction in learning rate (Vakil et al., 2010), the difference in scores between list A3 and list A1, and the recall-ratio, the ratio of mean scores on recall vs. acquisition trials (Benedict et al., 1998).

Not surprisingly, subjects with greater education show better VLT performance (Paolo et al., 1997; Benedict et al., 1998; Hester et al., 2004; Magalhães and Hamdan, 2010; Argento et al., 2015). There are also reliable sex differences: Women generally outperform men (Bolla-Wilson and Bleecker, 1986; Bleecker et al., 1988; Geffen et al., 1990; Wiens et al., 1994; Vanderploeg et al., 2000; Hayat et al., 2014; Lundervold et al., 2014; Argento et al., 2015).

The BAVLT includes additional measures of word retrieval latencies, serial positon effects, and the semantic organization of responses. Interword intervals (IWIs) have not been studied in the RAVLT, CVLT, or HVLT, but we expected that they would increase monotonically with serial recall position, as has been found in other list learning tasks (Wixted and Rohrer, 1994). We also expected to find an increase in mean IWIs in older participants, reflecting age-related slowing of word retrieval and response generation.

Serial position effects (Capitani et al., 1992) have received considerable attention in recent years because reduced primacy effects have been found to characterize the performance of patients with Alzheimer's disease (AD) (Bayley et al., 2000; Foldi et al., 2003) and are predictive of the conversion of mild cognitive impairment to AD (Egli et al., 2014). Moreover, reduced primacy effects are seen in cognitively normal individuals at risk for AD because of family history (La Rue et al., 2008). We anticipated that subjects with poorer BAVLT acquisition and recall scores would show reduced primacy effects.

Because the 12 words in the BAVLT lists came from four different semantic categories, we were also able to examine the sematic clustering of responses over successive acquisition and recall trials. We anticipated that semantic clustering would increase over successive list presentations and would be greater in recall than acquisition trials.

## Experiment 1: methods

### Ethics statement

Subjects in all experiments gave informed written consent following procedures approved by the Institutional Review Board of the Veterans Affairs Northern California Health Care System (VANCHCS) and were paid for their participation.

### Participants

We studied 195 control participants, whose demographic characteristics are included in Table 3. The participants ranged in age from 18 to 82 years (mean age = 40.3 years), had an average education of 14.7 years, and were native English speakers. Fifty-seven percent of the control participants were male. Participants were recruited from advertisements on Craigslist (sfbay.craigslist.org) and pre-existing control populations. They were required to meet the following inclusion criteria: (a) no current or prior history of psychiatric illness; (b) no current substance abuse; (c) no concurrent history of neurologic disease known to affect cognitive functioning; (d) on a stable dosage of any required medication; (e) auditory functioning sufficient to understanding normal conversational speech and (f) visual acuity normal or corrected to 20*/*40 or better. Participant ethnicities were 64% Caucasian, 12% African American, 14% Asian, 10% Hispanic/Latino, 2% Hawaiian/Pacific Islander, 2% American Indian/Alaskan Native, and 4% “other.”

**Table 3:** **Mean performance measures**.

	***N***	**Age**	**Sex**	**Edu**	**A1**	**A2**	**A3**	**A3-A1**	**B**	**IR**	**T Acq**	**DR**	**Omn**	**Omn z**	**Err**	**Recog**	**P/R**	**IWI**
Exp 1	**195**	**40.3** (20.2)	**0.57** (0.50)	**14.7** (2.10)	**5.61** (1.82)	**7.96** (2.03)	**8.97** (2.13)	**3.37** (1.68)	**6.12** (1.90)	**7.38** (2.66)	**22.54** (5.32)	**7.15** (2.69)	**43.18** (11.13)	**0.00** (1.00)	**5.18** (4.19)	**11.36** (1.15)	**1.11** (0.39)	**2.77** (1.27)
Exp. 2a	**29**	**26.38** (4.62)	**0.45** (0.51)	**15.0** (1.24)	**7.34** (1.52)	**9.86** (1.46)	**10.41** (1.12)	**3.07** (1.83)	**7.28** (1.81)	**9.00** (1.87)	**27.62** (3.08)	**8.76** (1.86)	**52.66** (7.39)	**0.54** (0.76)	**4.21** (3.36)	**11.66** (0.67)	**1.26** (0.68)	**2.62** (1.06)
Exp. 2b					**7.34** (1.61)	**9.52** (1.68)	**10.90** (1.18)	**3.55** (1.74)	**7.17** (1.77)	**10.03** (1.76)	**27.76** (3.60)	**9.83** (2.11)	**54.79** (7.07)		**2.31** (2.74)	**11.90** (0.31)	**0.99** (0.18)	**2.33** (0.73)
Exp. 2c					**7.93** (1.53)	**10.69** (1.34)	**11.03** (1.09)	**3.10** (1.57)	**7.45** (1.92)	**10.24** (1.43)	**29.66** (3.31)	**9.55** (2.32)	**56.90** (7.65)		**2.07** (2.19)	**11.76** (0.51)	**1.05** (0.19)	**2.45** (0.87)
Exp 3	**27**	**26.85** (4.65)	**0.48** (0.51)	**14.9** (1.25)	**5.52** (1.70)	**7.15** (2.01)	**7.37** (2.11)	**1.85** (1.49)	**5.19** (1.75)	**5.37** (2.15)	**20.04** (5.33)	**5.56** (2.10)	**36.15** (10.23)	**-1.19** (1.17)	**7.78** (6.02)	**9.37** (1.78)	**0.90** (0.45)	**3.84** (1.67)
mTBI	**24**	**33.71** (11.05)	**1.00** (0.00)	**13.5** (1.38)	**5.33** (1.81)	**7.38** (1.84)	**9.17** (1.95)	**3.83** (1.83)	**5.67** (1.71)	**7.04** (2.74)	**21.88** (4.90)	**6.71** (3.01)	**41.29** (10.86)	**0.02** (1.19)	**5.46** (4.26)	**11.04** (1.16)	**1.19** (0.52)	**2.88**. (0.92)
sTBI	**4**	**46.00** (8.98)	**0.75** (0.50)	**13.0** (1.15)	**3.75** (1.89)	**5.75** (1.26)	**7.75** (1.26)	**4.00** (0.82)	**4.75** (0.96)	**5.00** (1.83)	**17.25** (4.27)	**6.50** (2.08)	**33.50** (7.94)	**-0.53** (0.86)	**5.75** (6.95)	**10.67** (1.53)	**0.93** (0.31)	**4.31** (1.14)

*Sex, males, 1; A1, A2, A3, mean number correct on 1st, 2nd, or 3rd presentation of list A; A3-1, learning rate score, the difference between the 3rd and 1st list A presentations; B, number correct on list B; IR, immediate recall after list B; T Acq, total acquisition score, the sum of correct words on lists A1 to A3 and B; DR, delayed recall 30 min after list presentation; Omn, omnibus scores, summed across all trials; Omn z, omnibus z-score; Err, total errors across all recall trials; Recog, recognition accuracy for list A; P/R, Primacy/recency ratio; IWI, mean interword interval. Note that two participants in Exp. 2 did not return for simulated malingering testing in Exp. 3. Bold values indicate mean values*.

### Apparatus and stimuli

The BAVLT was the eighth test in the California Cognitive Assessment Battery (CCAB), and required 8 to 12 min per participant. Each CCAB test session included the following additional computerized tests and questionnaires: Finger tapping (Hubel et al., 2013a,b), simple reaction time (Woods et al., 2015a,d), Stroop, digit span forward and backward (Woods et al., 2011a,b), verbal fluency (Woods et al., 2016a), visuospatial span (Woods et al., 2015c,e), trail making (Woods et al., 2015b), vocabulary, design fluency (Woods et al., 2016b), the Wechsler Test of Adult Reading (WTAR), choice reaction time (Woods et al., 2015a,f), risk and loss avoidance, delay discounting, the Paced Auditory Serial Addition Task (PASAT) (Woods et al., under review), the Cognitive Failures Questionnaire (CFQ) and the Posttraumatic Stress Disorder Checklist (PCL)(Woods et al., 2015g), and a local traumatic brain injury (TBI) questionnaire. Testing was performed in a quiet room using a pair of headphones and a Windows computer controlled by Presentation® software (Versions 13 and 14, NeuroBehavioral Systems, Berkeley CA). An executable, open-source version of the BAVLT for Windows is available for download at http://www.ebire.org/hcnlab/.

### Procedure

In Experiment 1, the 12 words in List A were selected from four semantic categories, with List A presented three separate times (A1, A2, and A3). Participants were instructed to listen to a list of words and immediately recall as many words as possible in any order. On the fourth trial, a list of 12 new words (List B) was presented, with six words from two new semantic categories and six words from two semantic categories shared with List A. Participants were instructed to only report words from List B during that recall period.

During list presentations, words were delivered at the rate of one word every 1.93 s through headphones at calibrated intensities of 80 dB SPL. Tones signaled to the participant when list presentation was complete. There was no time limit on the recall period. However, after a pause of 30 s in which no responses were made, participants were asked “Do you remember any more words?”

After List B recall, subjects were asked to recall List A words during the immediate recall (IR) trial. After a 30-min delay filled with other neuropsychological tests, participants were instructed to recall as many words as possible from List A during a delayed recall (DR) trial followed by a visual, two-alternative forced-choice recognition task. Two words appeared on their computer monitor: One word was from List A, while the other word had not been presented in any List, but was in the same semantic category as the corresponding List A token. Participants selected the List A token with the mouse while accuracy and response times were recorded. Subjects required an average of 24.9 s to complete the BAVLT recognition test, much less time than required to complete recognition tests where subjects indicate whether each auditorily-presented word occurred in List A. Recognition scoring was also more objective because forced-choice testing eliminates the influence of criterion levels and response biases.

**Figure 1:**
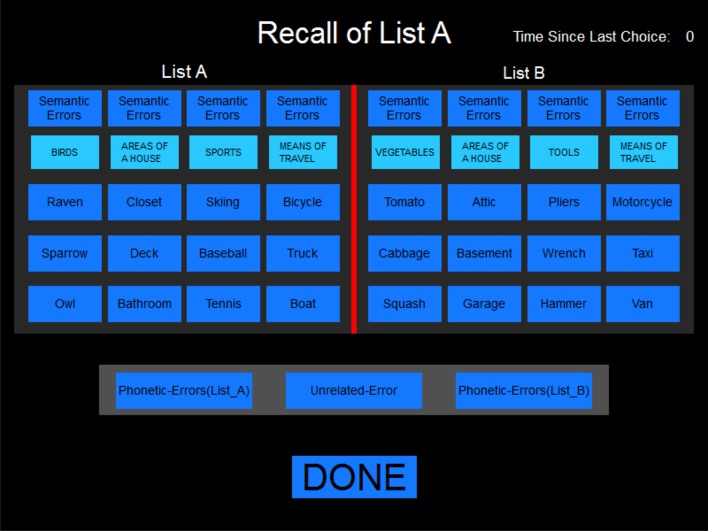
**The scoring display**. The display as it appeared to the examiner during the immediate free recall of List A and after the presentation of List B. Only List A tokens were displayed during the first three presentations of list A.

The examiner was seated in the test room behind and to the side of the participant and used the mouse to select words retrieved on the examiner screen (See Figure 1). The order of responses was recorded along with response latencies. The time required for subjects to complete the BAVLT averaged about 10 min, including test instructions, brief rest periods, and the completion and scoring of trials.

### Error scoring

Four separate types of errors were categorized using the on-screen examiner interface (see Figure 1): Semantic errors, words not on the list that belonged to one of the four semantic categories; phonetic errors, incorrect words that phonetically resembled one of the list words; intrusion errors, words reported from List A when instructed to recall List B, or vice versa; and unrelated errors. Repeated words were tallied and a total error score (including repetitions) was summed across all trials.

### Performance measures

The BAVLT tallied the number of words correctly recalled for each presentation in the acquisition phase (A1, A2, A3) and List B. A total acquisition score (the sum of correct words on trials A1, A2, and A3) was also obtained, along with recall scores on IR and DR trials. A retrieval ratio was obtained by dividing the average of the DR and IR scores by the average score on the three learning trials (A1, A2, and A3). The learning rate was measured with the A3–A1 difference score. Finally, an omnibus score was obtained: The sum of words recalled on acquisition, distractor, and recall trials.

Serial position functions, the percentage correct at each serial position, were analyzed for each trial. The average accuracy for the first three words in the list was used to estimate primacy effects, the accuracy of the middle six words was used to estimate mid-list performance, and the accuracy on the final three words was used to estimate recency effects. The average primacy/recency ratio was also analyzed for each participant, averaged over trials of all types.

### Analysis of semantic clustering

Each list included 12 words separated into four semantic categories (see Appendix A for the word lists), with one word from each semantic category in each successive group of four words. Word order was fixed for all trials and subjects. The degree to which recall contained words clustered into semantic categories was quantified using a category organization index (COI), which was the sum of the number of recalled words in the same semantic group as the previous word, normed by dividing by the maximal COI score possible given the number of words recalled. As a result, COI scores ranged from 0.0 (no clustering by semantic category) to 1.0 (all words clustered by semantic category). Repetitions could either break or continue a cluster (but not exceed the maximal cluster size), as could semantic errors. Unlike the semantic clustering measures of the CVLT (Stricker et al., 2002), COI measures were independent of the number of words recalled. For example, if a and b refer to words in two 3-word categories in the 12-word list, the recall of all three words in the one category (i.e., a-a-a) would have the same COI (1.0) as the recall of six clustered words, three from each of two different categories (i.e., a-a-a-b-b-b). In contrast, CVLT measures of semantic clustering in these two cases would be 1.64 and 6.00, respectively.

### Recall timing

We measured the onset latency (OL) to the first word recalled as well as the IWIs between subsequent words. These latency measures depended on the subject's response and the examiner's reaction time (RT) to log the response. We analyzed the RTs of one examiner using a program which presented List A words and distractors randomly. The examiner responded with a mean latency 0.88 s (0.32 s), with 95% of selections occurring in less than 1.46 s. Thus, the examiner RT represented a relatively small fraction of the OL (mean 5.13 s), and RT variance was also a small fraction of the average IWI (2.77 s).

### Statistical analysis

The results were analyzed with Analysis of Variance (ANOVA) using CLEAVE (www.ebire.org/hcnlab). Greenhouse-Geisser corrections of degrees of freedom were uniformly used in computing p values in order to correct for covariation among factors and interactions, with effect sizes reported as partial ω^2^. Pearson correlation analysis was also used with significance levels evaluated with Student's *t*-tests. Linear multiple regression was used to evaluate the contribution of multiple demographic factors on performance and to produce z-scores. Because of the large number of comparisons performed, alpha levels were set at 0.005.

Nine trained examiners administered the tests. However, three of the examiners administered 23 or more tests, and aggregately administered 73% of all tests. Inter-examiner differences were analyzed by comparing the scores of the participants tested by these three examiners.

## Results: Experiment 1

**Figure 2:**
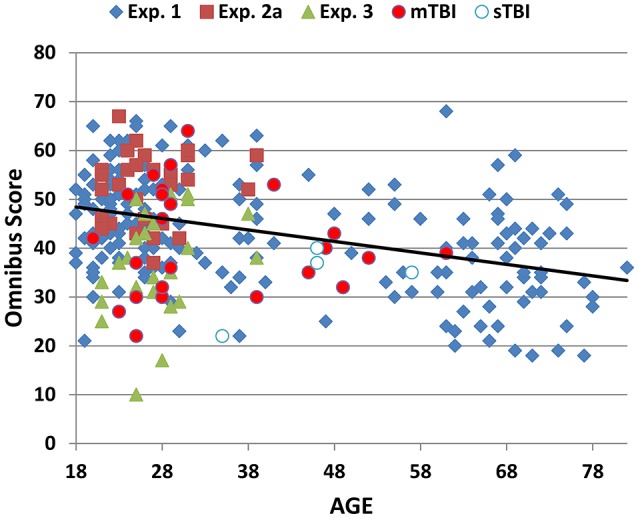
**Omnibus scores as a function of age**. Omnibus scores were the sum of correctly reported words during the presentation of four acquisition trials (including list B), and two recall trials (IR and DR). Data are shown for normative control subjects in Experiment 1 (blue diamonds), Experiment 2a (red squares), Experiment 3 (Malingering, green triangles), and Experiment 4 with separate data for patients with mild (red circles) and severe (blue and white) TBI.

Figure 2 show the omnibus scores of the participants in Experiment 1 (blue diamonds) and the other experiments (discussed below) as a function of age. Experiment 1 participants achieved mean acquisition scores of 22.54 (5.32) and omnibus scores of 43.2 (11.1). Table 3 provides summary performance statistics for acquisition, recall, and recognition trials in Experiment 1, as well as derived measures including omnibus scores, total acquisition scores, learning rate (A3–A1), total errors, and primacy/recency ratios.

**Figure 3:**
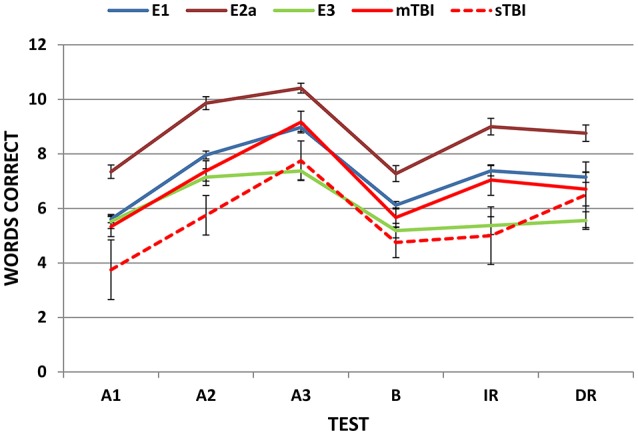
**Mean correct-word scores on successive trials**. E1, Experiment 1; E2a, Experiment 2a, E3, Experiment 3. Error bars show standard errors. Experiment 4 data are shown separately for patients with mild and severe TBI (mTBI and sTBI).

Figure 3 shows correct-word scores for the different lists in Experiment 1 (blue line). ANOVA analysis of Experiment 1 with Lists (A1, A2, A3, B, IR, DR) as a factor showed a highly significant main effect [*F*_(5, 970)_ = 157. 64, *p* < 0.0001, ω^2^ = 0.45] with significant differences (*p* < 0.02) in recall for each list, except for the IR/DR comparison. As seen in Figure 3, there was a steep increase in recall from lists A1 to A3, with a mean learning rate score of 3.37 (1.68), a predictable decline on List B, a small decline from List A3 to IR (1.59, sd = 1.77), and little further decline from IR to DR (0.23, sd = 1.37). Despite the fact that the list contained only 12 words, ceiling effects were relatively rare: 11.8% of subjects achieved perfect scores on List A3, 6.2% on IR, and 5.1% on DR trials.

The correlation matrix for Experiment 1 is shown in Table 4. Scores on lists A1, A2, and A3 were strongly correlated with each other and with IR and DR scores. Correlations increased across A1, A2, and A3 scores, and A3 scores were highly predictive of IR [*r* = 0.76, t_(193)_ = 16.25, *p* < 0.0001] and DR [*r* = 0.75, t_(193)_ = 15.75, *p* < 0.0001] scores.

**Table 4:** **Correlation matrix for Experiment 1**.

	**Sex**	**Edu**	**A1**	**A2**	**A3**	**A3-A1**	**B**	**IR**	**T Acq**	**DR**	**Omn**	**Omn z**	**Err**	**Recog**	**IWI**	**P/R**
Age	0.11	0.13	−0.34	−0.38	−0.44	−0.19	−0.21	−0.38	−0.44	−0.37	−0.43	0.00	0.27	−0.36	0.23	−0.20
Sex		−0.04	−0.20	−0.30	−0.27	−0.13	−0.10	−0.22	−0.29	−0.17	−0.25	0.00	0.03	−0.21	0.04	−0.07
Edu			0.16	0.20	0.19	0.07	0.25	0.19	0.21	0.25	0.25	0.00	−0.05	0.03	−0.06	0.08
A (1)				0.66	0.65	−0.26	0.49	0.62	0.86	0.64	0.79	0.67	−0.25	0.40	−0.13	0.17
A (2)					0.73	0.21	0.51	0.69	0.90	0.69	0.85	0.68	−0.27	0.37	−0.15	0.22
A (3)						0.56	0.46	0.76	0.90	0.75	0.87	0.69	−0.27	0.47	−0.07	0.23
A3–1							0.06	0.30	0.22	0.25	0.25	0.14	−0.06	0.16	0.05	0.10
B								0.50	0.55	0.47	0.66	0.68	−0.11	0.21	−0.16	0.18
IR									0.78	0.87	0.91	0.77	−0.29	0.42	−0.19	0.24
Acq										0.78	0.95	0.77	−0.29	0.47	−0.13	0.23
DR											0.90	0.77	−0.27	0.47	−0.12	0.22
Omn												0.83	−0.29	0.47	−0.16	0.25
Omn z													−0.18	0.32	−0.04	0.15
Err														−0.22	0.33	−0.06
Recog															−0.03	0.28
IWI																0.01

*See Table 3 for additional abbreviations. Given the number of observations (n = 195), r > |0.19| is significant at p < 0.01; r > |0.24| is significant at p < 0.001; and r > |0.28| is significant at p < 0.0001 without Bonferroni correction*.

**Figure 4:**
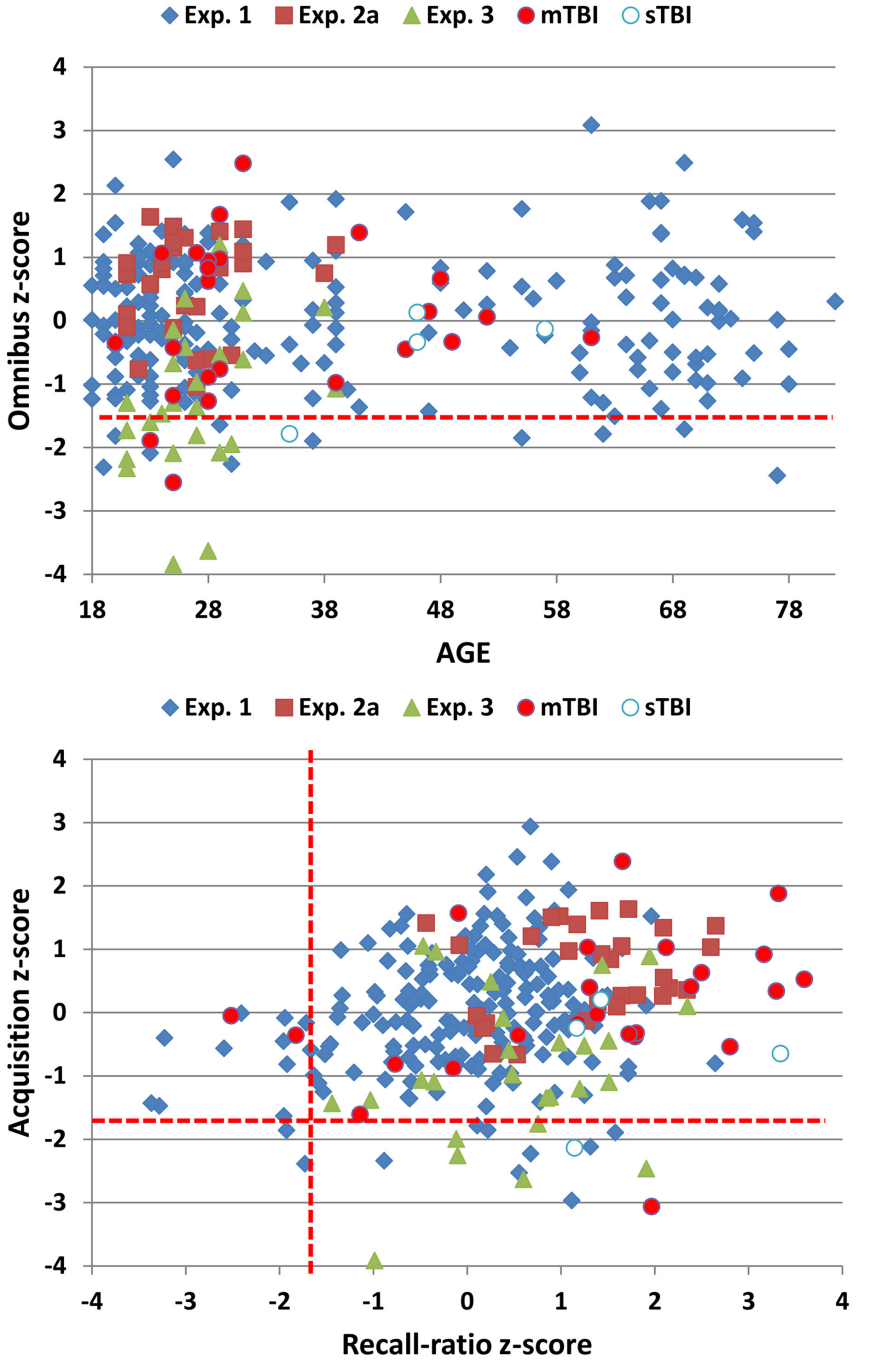
**Top:** Omnibus z-scores as a function of age. **Bottom:** Acquisition vs. recall-ratio z-scores. Omnibus and acquisition scores were transformed into z-scores after factoring out the contributions of age, sex, and education; recall-ratio z-scores factored out age and education. See Figure 2 for details. The dashed red lines show *p* < 0.05 abnormality thresholds for Experiment 1.

Omnibus scores correlated significantly with age [*r* = −0.42, t_(193)_ = −6.62, *p* < 0.0001], education [*r* = 0.25, t_(193)_ = 3.59, *p* < 0.0004], and sex (males = 1, females = 0): Women had greater omnibus scores than men [*r* = −0.25, t_(193)_ = 3.59, *p* < 0.0004]. Multiple regression analysis with Age, Education, and Sex as factors accounted for 31% of the variance in omnibus scores and was used to calculate predicted scores using the equation: 32.69–0.245^*^Age −4.22^*^Sex + 1.55^*^ Education. Omnibus z-scores, corrected for age, education, and sex, are shown for Experiment 1 participants (blue diamonds) as a function of age in Figure 4 (top).

Two other z-scores were also obtained (Figure 4, bottom). Like omnibus scores, total acquisition scores (the sum of words retrieved in A1, A2, and A3) correlated significantly with age, sex, and education. Multiple regression analysis with these three factors accounted for 32% of variance, and was used to calculate a predicted acquisition z-score using the equation 19.45–0.117^*^Age −2.483^*^Sex + 0.629^*^ Education. The recall-ratio, the ratio of the average number of words recalled in IR and DR trials relative to acquisition trials, was used to evaluate forgetting. The recall-ratio was not significantly influenced by sex, but showed significant influences of age and education. Multiple regression with Age and Education as factors accounted for 7% of recall-ratio score variance, and was used to calculate recall-ratio z-scores using the equation 0.642–0.002^*^Age + 0.013^*^ Education. We found that acquisition z-scores and recall-ratio z-scores showed a weak but borderline significant correlation [*r* = 0.19, t_(193)_ = 3.90, *p* < 0.008].

Age was associated with declines in recognition scores [*r* = −0.36, t_(193)_ = −5.36, *p* < 0.0001], and older participants showed a trend toward reduced learning rates [*r* = −0.19, t_(193)_ = 3.90, *p* < 0.008]. In addition, total errors increased with age [*r* = 0.27, t_(193)_ = 3.90, *p* < 0.0002], as seen in Table 4. This was due primarily to an age-related increase in phonemic errors [*r* = 0.49, t_(193)_ = 7.81, *p* < 0.0001], which likely reflected hearing impairments in some of the older subjects, although unrelated errors and intrusion errors also showed a trend toward age-related increases [*r* = 0.17, t_(193)_ = 3.90, *p* < 0.02 for both comparisons]. Repetition errors did not increase with age [*r* = −0.02, NS].

### Response timing

Onset latencies and mean IWIs are provided in Table S1 for the different trial types in Experiment 1. Mean IWIs increased with age [*r* = 0.23, t_(193)_ = 3.28, *p* < 0.002], but age did not significantly affect OLs [*r* = 0.04. NS]. Increased OLs were associated with reduced omnibus scores [*r* = −0.25, t_(193)_ = 3.59, *p* < 0.0005] and a similar trend was seen for IWIs [*r* = −0.16, t_(193)_ = 2.25, *p* < 0.03]. Increased IWIs were also associated with an increase in errors [*r* = 0.33, t_(193)_ = 4.86, *p* < 0.0001].

ANOVA analysis of OLs showed that they were significantly influenced by Trial type [*F*_(5, 955)_ = 18.88, *p* < 0.0001, ω^2^ = 0.09]: OLs were reduced on acquisition trials compared to recall trials (*p* < 0.0001). Onset latencies were also longer on IR than DR trials (*p* < 0.0001), possibly reflecting proactive interference from List B presentation. Mean IWIs also showed a significant effect of Trial [*F*_(5, 930)_ = 14.46, *p* < 0.0001, ω^2^ = 0.07] due to longer mean IWIs on A1 than subsequent List A presentations [*p* < 0.05 to 0.004], and longer IWIs on recall than acquisition trials (*p*<0.0001).

**Figure 5:**
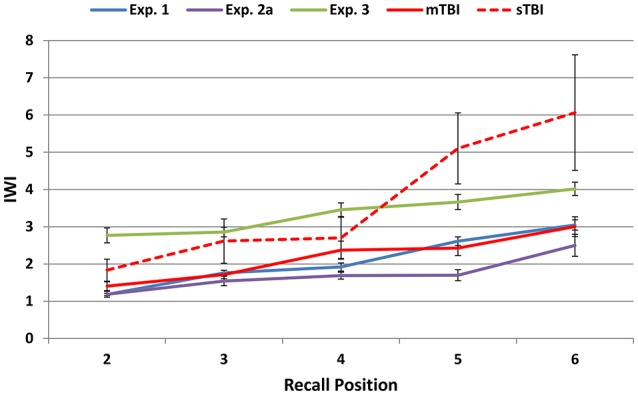
**Mean inter-word intervals (IWIs) for words 2 through 6 in the different experiments**. The data were averaged over trials of different types and output lengths. See Figure 2 for abbreviations.

Figure 5 shows mean IWIs averaged across all trial types for responses 2 to 6: Mean IWIs showed a largely linear increase from 1.1 s on response 2 to more than 3.0 s on response 6. IWIs increased with each successive word recalled, as did IWI dispersion. The increased dispersion was largely due to occasional long IWIs that occurred late in the recall sequences and were often associated with errors. Table S2 shows IWIs for the subset of list reports that contained eight words: Large increases in IWIs for later responses were accompanied by corresponding increases in IWI standard deviations and error rates.

### Serial position effects

**Figure 6:**
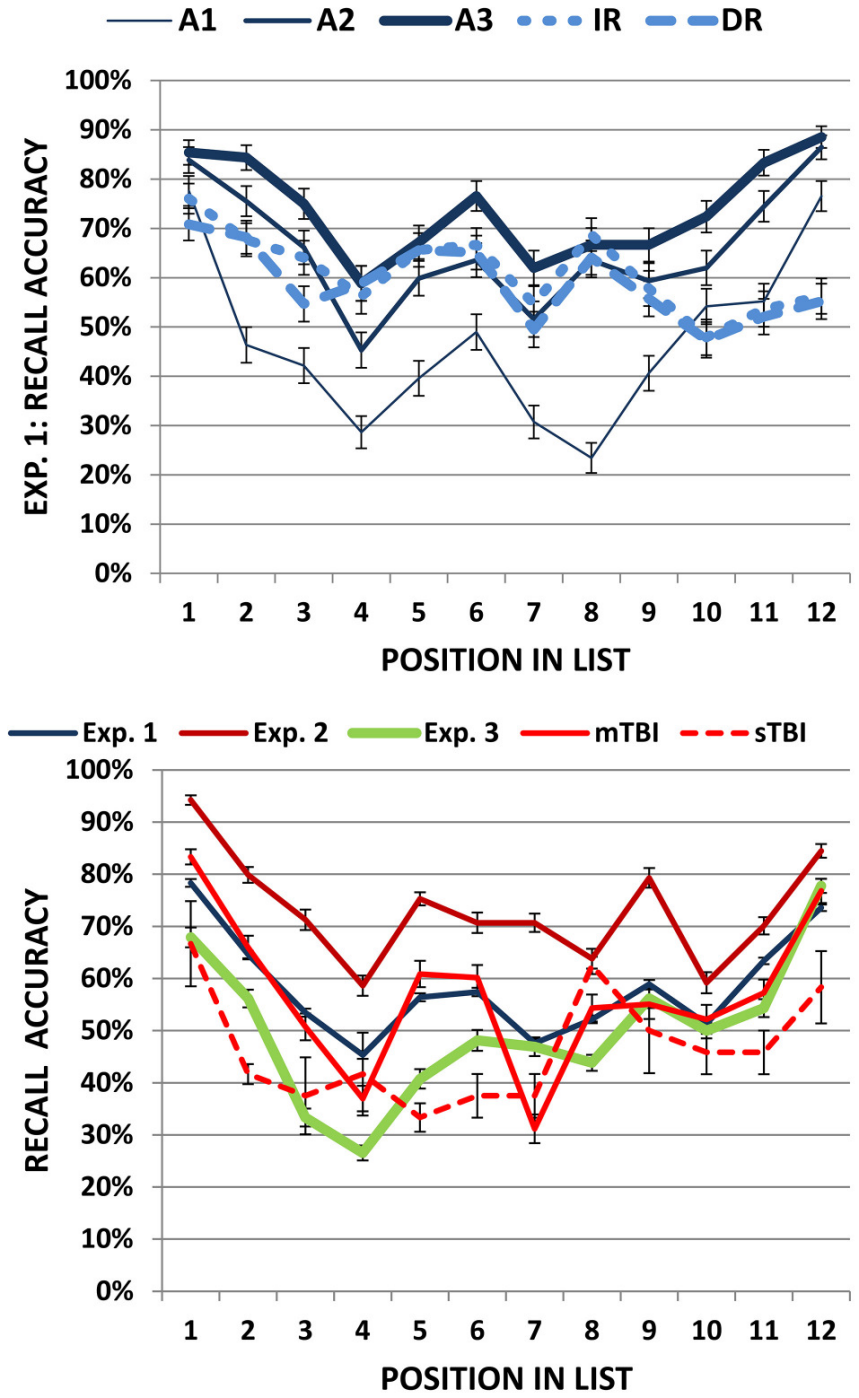
**Top:** Serial position functions in Experiment 1 shown separately for learning (A1, A2, and A3) and recall trials (IR and DR). **Bottom:** Serial position functions averaged over learning and recall trials for all experiments. Error bars show standard errors. See Figure 2 for abbreviations.

Figure 6 (top) shows the percentage of words correctly recalled as a function of serial position for the three acquisition (solid lines) and two recall (dashed lines) trials. ANOVA for repeated measures was used to evaluate primacy and recency effects during acquisition with Trial (A1 to A3) and list Position (primacy, mid-list, or recency) as factors. The Trial main effect was highly significant [*F*_(2, 382)_ = 372.36, *p* < 0.0001, ω^2^ = 0.66] due to increases in recall accuracy between A1 and A2 (*p* < 0.0001) and A2 and A3 (*p* < 0.0001). The Position factor was also significant [*F*_(2, 382)_ = 73.02, *p* < 0.0001, ω^2^ = 0.27] due to the superior recall of primacy (71.3% correct) and recency (73.1% correct) tokens compared to mid-list tokens (53.5% correct). Finally, there was a Trial x Position interaction [*F*_(4, 764)_ = 4.08, *p* < 0.005, ω^2^ = 0.02] that reflected greater improvement for mid-list tokens from List A1 to A3 (+32%) than for primacy (+27%) or recency (+20%) tokens. An additional ANOVA comparing lists A1 and B showed the expected Position effect [*F*_(2, 382)_ = 68.22, *p* < 0.0001, ω^2^ = 0.26] without a significant Trial x Position interaction [*F*_(2, 382)_ = 1.28, NS]; i.e., Lists A1 and B showed similar primacy and recency effects.

A comparison of List A3 and IR showed significant Trial [*F*_(1, 191)_ = 185.92, *p* < 0.0001, ω^2^ = 0.49] and Position [*F*_(2, 382)_ = 21.10, *p* < 0.0001, ω^2^ = 0.10] effects, and a strong Trial x Position interaction [*F*_(2, 382)_ = 44.39, *p* < 0.0001, ω^2^ = 0.19]. Compared to List A3, IR trials showed a greater decrement for recency tokens (−29.5%) than for mid-list (−5.5%) or primacy (−13%) tokens (Jahnke, 1968). A comparison of IR and DR trials did not show significant additional forgetting over the 30-min delay interval [*F*_(1, 191)_ = 2.10, *p* < 0.15]. There was a significant Position effect on recall trials [*F*_(2, 382)_ = 29.78, *p* < 0.0001, ω^2^ = 0.13] due to better recall of primacy (67.6% correct) and mid-list (61.0% correct) tokens than recency (52.7% correct) tokens, without a significant Trial x Position interaction [*F*_(2, 382)_ = 1.23, NS].

The blue line in the bottom panel of Figure 6 shows the mean serial position function averaged over acquisition, distractor, and recall trials for Experiment 1 participants. The mean primacy/recency ratio averaged over all trials was 1.11 (sd = 0.39). The primacy/recency ratio increased in parallel with omnibus scores [*r* = 0.25, t_(189)_ = 3.55, *p* < 0.0005] and, consistent with previous results (Capitani et al., 1992; Golomb et al., 2008), showed a strong trend toward decline with increasing age [*r* = −0.20, t_(189)_ = −2.81, *p* < 0.006].

### Semantic organization of recall

Category organization index (COI) values for the different trials of Experiment 1 are shown in Table S3. There was little evidence of semantic re-organization in Experiment 1: COIs were low (mean 0.25) and uniform across the successive acquisition and recall trials [*F*_(5, 935)_ = 1.34, NS], and they did not correlate significantly with omnibus z-scores, acquisition z-scores, or recall-ratio z-scores.

### Inter-examiner differences

We analyzed scores from the three examiners who respectively administered 23, 28, and 95 tests, and found no significant examiner effects on omnibus z-scores [*F*_(2, 139)_ = 1.27, NS], acquisition z-scores [*F*_(2, 139)_ = 1.14, NS], recall-ratio z-scores [*F*_(2, 139)_ = 0.39, NS], or mean IWIs [*F*_(2, 139)_ = 1.42, NS].

## Experiment 1. discussion

The influences of age, sex, and education on BAVLT performance were similar to those observed with the RAVLT (Geffen et al., 1990; Magalhães and Hamdan, 2010; Speer et al., 2014), the CVLT (Wiens et al., 1994; Kramer et al., 2003; Woods et al., 2006), and the HVLT (Brandt, 1991; Benedict et al., 1998; Vanderploeg et al., 2000). Both acquisition and recall scores declined with increasing age. As a result, the oldest participants had omnibus scores that were about 1.2 standard deviations below the mean scores of the youngest participants. Older participants also showed decreased IR and DR scores, and a slight decrease in the recall-ratio; i.e., they tended to forget a higher percentage of words that had previously been recalled. Women outperformed men by about 0.5 standard deviations, and participants with a college or post-college education outperformed participants with a high-school education by about 0.7 standard deviations.

The BAVLT did not show a significant examiner influence of the sort that has been reported in previous studies (Wiens et al., 1994; Van Den Burg and Kingma, 1999). Three factors likely contributed to reduced examiner influences. First, word presentation was acoustically controlled, with identical tokens delivered at calibrated intensities and constant IWIs on each list presentation. Second, the experimenter sat slightly behind the subject and remained outside the subject's field of view. This minimized visual and social cues. Third, response recording was facilitated by the scoring screen, and response logging and analysis was performed automatically, reducing the potential errors found in manual scoring, transcription, and analysis.

Ceiling effects can influence VLT metrics by limiting A3 recall and A3–A1 learning-rate scores. Since ceiling effects occur disproportionately among younger participants, they can also confound the analysis of age-related changes in learning and forgetting (Uttl, 2005). Ceiling effects were less prominent on the BAVLT than the HVLT (see Table 2). For example, the mean recall score on BAVLT List A3 (8.97 words, standard deviation = 2.13 words) was 1.42 standard deviations below maximal scores (i.e., 12), while mean recall scores on List A3 of the HVLT are often less than one standard deviation below ceiling scores (Brandt, 1991; Benedict et al., 1998). The RAVLT and CVLT present, respectively, 15- and 16-word lists on five acquisition trials. Based on the means and standard deviations of scores on trial A5, ceiling effects on the BAVLT would appear to be somewhat more common than on the CVLT (Woods et al., 2006), but somewhat less common than on the RAVLT (Uttl, 2005; Magalhães and Hamdan, 2010).

In all tests, the correlation between List A scores increases across acquisition trials. For example, the correlation between A1 and A2 was 0.66, which increased to 0.73 between trials A2 and A3. Not surprisingly, correlations are even higher for later acquisition trials with five list A presentations, as on the CVLT and RAVLT. For example, RAVLT scores on lists A3 and A4 show a correlation of 0.80, and scores on lists A4 and A5 show a correlation of 0.81 (Magalhães and Hamdan, 2010). These results suggest that the presentation of Lists A4 and A5 provides limited additional information in comparison with the three list presentations used in the HVLT and BAVLT.

### Retrieval latencies

Although the time needed to retrieve words from long-term memory distinguishes patients with Alzheimer's disease from controls in verbal fluency tests (Rohrer et al., 1995), retrieval latencies have not been examined on the RAVLT, HVLT, or CVLT. The analysis of retrieval latencies on the BAVLT showed predictable differences across lists. Onset latency measures were relatively short for acquisition trials and significantly prolonged for the IR trial, presumably reflecting the additional time needed for participants to segregate the words in List A from the words in List B. Mean IWIs declined over acquisition trials A1 to A3 as participants became more familiar with the list, and then increased during immediate and delayed recall. As in previous studies (Rohrer, 1996), IWIs for individual words increased with serial recall position, and words preceded by long IWIs showed increased error rates.

### Serial position effects

Consistent with prior studies (Suhr, 2002; Powell et al., 2004; Gavett and Horwitz, 2012; Egli et al., 2014), we found strong primacy and recency effects on initial list presentations (A1 and B) that diminished with learning. Recency effects were absent on recall, consistent with suggestions that recency effects depend on access to a short-term buffer that is overwritten by the presentation of competing lists (Davelaar et al., 2005). We also found a strong trend (*p* < 0.006) for the primacy/recency ratio to decline with age.

### Semantic organization

We did not find support for our hypothesis that semantic organization would increase over successive trials. Two factors may have contributed to the relatively low levels of semantic organization observed. First, the semantic grouping was relatively subtle, since each semantic group contained only three related words. Second, *post-hoc* analysis showed that some of the words in List A showed strong, across-group semantic associations. For example, “deck” (an area of the house, see Appendix A) and “boat” (a means of transportation) were strongly associated. Such cross-category associations would reduce the magnitude of apparent semantic reorganization by recall category.

## Experiment 2: test-retest reliability

A number of studies have examined the test-retest reliability of VLTs. Test-retest reliabilities are generally high when the same test is presented repeatedly, with scores increasing over tests due to a combination of content learning (i.e., learning the words) and procedural learning (i.e., learning test procedures). For example, the meta-analysis of Calamia et al. (2013) found mean Pearson correlation coefficients of 0.74 for total acquisition scores, 0.63 for IR scores, and 0.88 for DR scores with RAVLT testing at intervals of 3 to 9 months. When different RAVLT lists were used, Calamia et al. (2013) found that the correlations declined to 0.67, 0.61, and 0.63, respectively. Lower test-retest reliability is generally reported for the HVLT, perhaps because HVLT lists are shorter and involve fewer list presentations. Although Benedict et al. (1998) found test-retest correlations of 0.74 and 0.66 on total acquisition and DR trials using different HVLT lists, other investigators have found lower test-retest correlations (Rasmusson et al., 1995; Barr, 2003; Woods et al., 2005).

Significant learning may also occur when identical lists are repeated. For example, Duff et al. (2001) tested 30 young control participants with identical CVLT lists at an average interval of 15.8 days, and found an increase in List A total acquisition scores of 23%, along with test-retest correlations of 0.55 for total acquisition scores, 0.47 for List B, 0.49 for IR, and 0.67 for DR. Woods et al. (2006) examined test-retest reliabilities in 195 participants at weekly intervals using identical CVLT-II lists and found Spearman's ρ correlations of 0.80 on List A total acquisition scores, 0.80 on IR scores, and 0.83 on DR scores. The respective correlations were reduced to 0.73, 0.67, and 0.69 when alternate lists were used. Other CVLT measures showed lower test-retest correlations, ranging from 0.06 for forced choice recognition to 0.63 for Trial 5 recall.

In Experiment 2, we anticipated high intraclass correlation coefficients (ICCs) for omnibus scores and acquisition measures, somewhat lower ICCs for individual trial scores and IWIs, and reduced ICCs for recognition scores (which were near ceiling levels for most participants), COIs, errors, and derived measures including the recall-ratio, learning rate scores, and the primacy/recency ratio. We also anticipated that scores would improve slightly over test sessions as subjects became more familiar with the test.

## Methods: Experiment 2

### Participants

The test administration methods were identical to those described in Experiment 1. Twenty-nine young volunteers (mean age 26.4 years, range 21 to 39 years, 45% male) were recruited primarily from online advertisements on Craigslist. Participants, who met the same inclusion criteria listed in Experiment 1, volunteered to participate in three weekly test sessions. Most participants were college or junior college students who were younger (*p* < 0.01) than the participants in Experiment 1, as seen in Table 3. Ethnically, 68% of the participants were Caucasian, 11% Latino, 9% African American, 10% Asian, and 2% other.

### Procedures

Experiment 2a used the same word lists as Experiment 1. Different word lists and semantic categories were used in Experiments 2b and 2c (see Appendix A). The order of the list presentation was identical for every participant.

### Statistical analysis

The results were analyzed with the same methods used in Experiment 1, with the addition that ICCs were analyzed with SPSS (version 22).

## Results: Experiment 2

**Figure 7:**
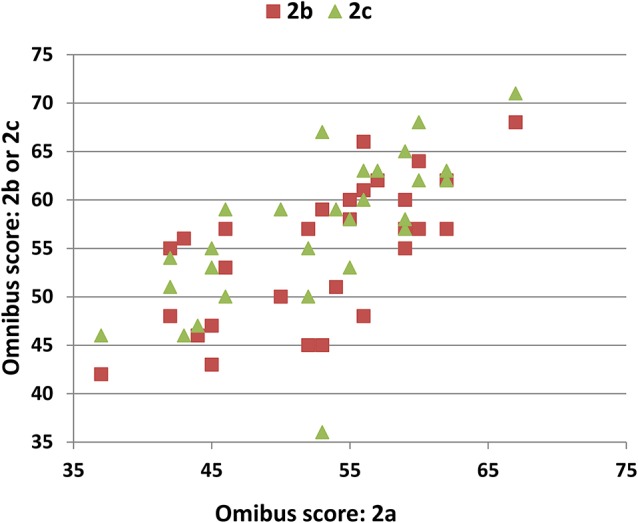
**Test-retest reliability**. Omnibus scores of the participants in Experiment 2a (abscissa) vs. Experiment 2b or 2c (ordinate).

Scatter plots of omnibus scores from the individual participants in Experiments 2a, 2b, and 2c are shown in Figure 7. Experiment 2a participants performed better than those in Experiment 1 in each phase of the experiment (see Table 3). In part, the higher scores reflected demographic differences: Experiment 2a participants were younger, slightly better-educated, and included a higher percentage of females. After factoring out the contributions of age, education, and sex using the regression equations from Experiment 1, omnibus z-scores still tended to be higher than those of Experiment1 participants [mean = 0.54, *F*_(1, 222)_ = 7.83, *p* < 0.006, ω^2^ = 0.03], and acquisition z-scores were significantly higher [*F*_(1, 222)_ = 11.89, *p* < 0.001, ω^2^ = 0.05]. As shown in Figure 3 (dark red line), scores were higher for all trial types.

Participants in Experiment 2 had better recall at all serial positions than the normative control group, with the largest differences seen for mid-list items (see Figure 6, bottom). However, the primacy/recency ratio (mean 1.26) was not significantly different from that obtained in Experiment 1 [*F*_(1, 215)_ = 3.05, *p* < 0.10]. Moreover, there were no significant differences in total recognition scores [*F*_(1, 222)_ = 1.84, NS], total error scores [*F*_(1, 222)_ = 1.44,NS], mean IWIs [*F*_(1, 222)_ = 0.54, NS], or recall-ratio z-scores [*F*_(1, 222)_ = 0.42, NS].

### Semantic organization

COI scores averaged 0.32 and tended to be higher than in Experiment 1 [*F*_(1, 222)_ = 6.64, *p* < 0.02, ω^2^ = 0.02]. Unlike Experiment 1, the COI scores in Experiment 2a tended to increase over successive trials [*F*_(5, 140)_ = 2.90, *p* < 0.03, ω^2^ = 0.06], with the highest level of semantic organization seen on DR trials (see Table S3). Finally, COI scores increased significantly from Experiment 2a to Experiment 2c [*F*_(2, 56)_ = 6.46, *p* < 0.002, ω^2^ = 0.16], reflecting increased semantic organization of recall as the participants became more experienced with the semantic organization of BAVLT lists.

### Procedural learning

Participants' omnibus scores increased by about 4.5% over each successive test (Table 3). ANOVA analysis of omnibus scores revealed a significant effect of test [*F*_(2, 56)_ = 7.44, *p* < 0.002, ω^2^ = 0.19], with trends toward significant differences between Experiment 2a and 2b (*p* < 0.01) and Experiment 2b and 2c (*p* < 0.01).

### Test-retest reliability

The ICCs for the different measures are shown in Table 5. The highest ICCs were seen for omnibus scores (0.86) and COIs (0.85). Omnibus z-scores and acquisition z-scores both had ICCs of 0.82, while the ICCs for IWIs, IR, and DR scores were above 0.75. ICCs were 0.58 for A1 recall and recall-ratio z-scores, and 0.60 for A2 and List B recall. Recognition, total-error scores, and learning-rate scores (A3–A1) showed lower ICCs (<0.32), as did derived scores including the primacy/recency ratios.

**Table 5:** **Reliability of BAVLT measures**.

	**Omni**	**Omn-z**	**A (1)**	**A (2)**	**A (3)**	**A3-A1**	**B**	**IR**	**DR**	**Err**	**Rec**	**IWI**	**P/R**	**T Acq z**	**Rec r z**	**COI**
ICC	0.86	0.82	0.53	0.60	0.76	0.28	0.60	0.76	0.76	0.32	0.10	0.77	0.25	0.82	0.58	0.85

*Intraclass correlation coefficients (ICCs) are shown for the different measures obtained in Experiment 2. Acq z, Acquisition z-score; Rec r z, recall-ratio z-score; See Table 3 for additional abbreviations*.

## Discussion: Experiment 2

The differences in omnibus and acquisition z-scores with the Experiment 1 population suggests that the regression functions in Experiment 1 did not fully capture all relevant factors contributing to BAVLT performance. Part of the improved performance of Experiment 2 participants was apparently due to increased semantic awareness: Their COI scores tended to be higher than those of Experiment 1 participants and increased across successive trials and test sessions.

Participant scores improved somewhat across test sessions despite the use of different word lists. Small improvements of similar magnitude have been seen for successive tests with different lists on the HVLT (Benedict et al., 1998) and CVLT (Woods et al., 2006). In Experiment 2, subjects increasingly recognized that words fell into different semantic categories, resulting in a significant increase in the COI scores across tests. However, because the equivalent difficulty of the different word lists had not been independently established, intrinsic differences in word-list difficulty and organization may have contributed to these apparent learning effects.

ICCs were acceptably high (>0.75) for most core measures, including omnibus z-scores, acquisition z-scores, COIs, IR and DR scores, and IWIs, and were somewhat reduced for recall-ratio z-scores (0.58). As with other verbal learning tests (Woods et al., 2005, 2006), reliabilities were considerably lower for learning rate measures, errors, recognition scores, and primacy/recency ratios.

The ICCs for most measures in the current study exceeded corresponding test-retest correlations of the RAVLT (Lemay et al., 2004; Rezvanfard et al., 2011; Calamia et al., 2013), the CVLT (Duff et al., 2001; Woods et al., 2006; Calamia et al., 2013), and the HVLT (Benedict et al., 1998; Barr, 2003; Woods et al., 2005). Additional measures also showed higher test-retest reliabilities than corresponding measures on the other tests. For example, COI reliability exceeded the test-retest reliabilities of all of the semantic clustering measures on the CVLT, including semantic clustering, serial clustering forward, serial clustering backward, and subjective clustering (Woods et al., 2006). In part, this may reflect the fact that our participants underwent three test sessions at short (1 week) test-retest intervals, whereas most previous studies examined test-retest correlations in only two sessions typically at longer test-retest intervals.

## Experiment 3: the effects of simulated malingering

Clinicians must often evaluate whether abnormal scores on neuropsychological tests are due to malingering or organic causes. Previous studies have shown VLT performance deficits in participants instructed to simulate malingering (Suhr and Gunstad, 2000; Suhr, 2002) and patients suspected of malingering (Boone et al., 2005; Curtis et al., 2006).

In Experiment 3, we investigated the sensitivity and specificity of malingering-detection procedures in discriminating participants with abnormal omnibus z-scores due to malingering from subjects in Experiment 1 with abnormal omnibus z-scores. We used four indices: (1) *Recognition scores*. Abnormal reductions in recognition scores have previously been observed in malingering populations (Sullivan et al., 2002; Whitney and Davis, 2015). (2) *Primacy/recency ratios*. Malingering participants show an abnormal reduction in primacy effects (Bernard et al., 1993; Suhr, 2002; Sullivan et al., 2002). (3) *Reduced learning rates*. We anticipated that simulated malingerers would show reduced learning rates (i.e., smaller A3-A1 difference scores) as they attempted to limit the number of words correctly recalled on late acquisition and recall trials. (4) *Increased IWIs*. We anticipated that simulated malingerers would show increased IWIs, particularly early in the recall period, because they would need to implement a more complex partial recall strategy rather than automatically recalling all of the words that came to mind. We used IWIs rather than OLs because OLs showed considerable intersubject variability in the normative population of Experiment 1 (range 1.96 to 17.77 s); i.e., some subjects appeared to mentally rehearse the entire list before beginning to report words, whereas others produced words as soon as the report period began.

## Methods: Experiment 3

### Participants

The participants were identical to those of Experiment 2, except that two of the 29 participants failed to return for the malingering test session.

### Materials and procedures

The methods and procedures were identical to those of Experiments 1 and 2a. However, after the third session of Experiment 2, participants were given written instructions to feign the symptoms of a patient with mild TBI during a test session the following week. The instructions were as follows: “Listed below you'll find some of the symptoms common after minor head injuries. Please study the List Below and develop a plan to fake some of the impairments typical of head injury when you take the test. Do your best to make your deficit look realistic. If you make too many obvious mistakes, we'll know you're faking! Symptom list: Difficulty concentrating for long periods of time, easily distracted by unimportant things, headaches and fatigue (feeling “mentally exhausted”), trouble coming up with the right word, poor memory, difficulty performing complicated tasks, easily tired, repeating things several times without realizing it, slow reaction times, trouble focusing on two things at once.”

### Statistical analysis

The results were analyzed using Analysis of Variance (ANOVA) between groups to compare the results with those of the normative controls in Experiment 1, and ANOVA within groups to compare the results to those in Experiment 2a. Other procedures were identical to those of Experiment 1.

## Results: Experiment 3

Omnibus scores from individual participants in Experiment 3 are included in Figure 2 (green triangles). Mean scores of simulated malingerers on the different trials are shown in Figure 3 (green line). Scores were similar to those of Experiment 1 subjects on list A1, but showed less improvement on list A2 and virtually no further improvement from list A2 to list A3.

The z-scores of the simulated malingerers were reduced (mean = –1.19), as shown in Figure 4. Relative to their performance in Experiment 2a, simulated malingerers showed reduced omnibus scores [*F*_(1, 26)_ = 67.90, *p* < 0.0001, ω^2^ = 0.72], reduced acquisition scores [*F*_(1, 26)_ = 32.06, *p* < 0.0001, ω^2^ = 0.54], and lower recognition scores [*F*_(1, 26)_ = 43.23, *p* < 0.0001, ω^2^ = 0.62]. There were also significant increases in errors [*F*_(1, 26)_ = 10.18, *p* < 0.005, ω^2^ = 0.26], mean IWIs [*F*_(1, 26)_ = 15.13, *p* < 0.0006, ω^2^ = 0.35], and OLs [*F*_(1, 26)_ = 13.50, *p* < 0.002, ω^2^ = 0.32].

Compared to the normative controls in Experiment 1, the simulated malingering group also showed reduced omnibus z-scores [*F*_(1, 219)_ = 31.95, *p* < 0.0001, ω^2^ = 0.12], acquisition z-scores [*F*_(1, 219)_ = 67.00, *p* < 0.0001, ω^2^ = 0.71], and recall-ratio z-scores [*F*_(1, 219)_ = 16.03, *p* < 0.0001, ω^2^ = 0.06]. Recognition scores were also markedly reduced [*F*_(1, 219)_ = 60.92, *p* < 0.0001, ω^2^ = 0.21]. Overall, 37% of the malingering group (10 simulated malingerers) had abnormal (*p* < 0.05) omnibus z-scores and 52% had abnormal recognition scores.

However, although ten simulated malingerers showed abnormal omnibus z-scores (*p* < 0.05), only two malingering participants showed z-scores below −3.00, i.e., clearly outside the range seen in control participants. Thus, z-score cutoffs were relatively ineffective at discriminating among subjects with abnormal scores (e.g., z-score < −3.00, sensitivity = 20%; specificity = 100%). We therefore examined other performance metrics of the 10 malingerers and 10 control participants with abnormal omnibus z-scores. We examined recognition scores, primacy/recency ratios, IWIs, and learning scores. Recognition scores were abnormal in 70% of malingerers and 20% of the controls with abnormal omnibus z-scores.

Figure 6 (bottom) shows the serial position functions for the simulated malingering group (green line). Consistent with previous reports (Suhr, 2002; Sullivan et al., 2002; Powell et al., 2004), primacy/recency ratios were abnormal (*p* < 0.05) in 60% of the simulated malingerers and 20% of the abnormal controls.

Figure 5 shows the IWIs by recall position for the malingering group (green line). Mean IWIs (see Table S1) were significantly prolonged relative to Experiment 1 across the list [*F*_(1, 212)_ = 15.04, *p* < 0.0001, ω^2^ = 0.06], with the largest difference, a 250% increase, seen in recall position 2. Overall, 60% of the abnormal malingerers showed an abnormal prolongation in the IWI for word 2 (i.e., at the beginning of the recall period), a pattern that was never observed among the abnormal control participants. Finally, learning rate scores were abnormal in 70% of abnormal malingerers vs. 30% of the abnormal controls.

Overall, 20% of abnormal malingerers showed abnormalities on all four indices, 40% showed three abnormalities, 20% showed two abnormalities, and 20% one abnormality. No malingering subjects showed no abnormalities. For the controls, 40% showed no abnormalities, 40% one abnormality, 10% two abnormalities and 10% three abnormalities. Thus, a cutoff of two or more abnormal criterion measures showed 80% sensitivity and 80% specificity in distinguishing abnormal scores due to simulated malingering from abnormal scores in control conditions. Moreover, of the 17 simulated malingerers whose omnibus z-scores remained within the normal range, 35% showed two or more abnormalities in the malingering-detection indices. In contrast, of the 183 control participants with normal omnibus z-scores, only 1.1% showed two or more abnormalities. Hence, the malingering-detection indices appear useful in identifying subjects with abnormal scores due to malingering, and may even assist in identifying malingering in participants whose omnibus z-scores fall within the normal range.

## Discussion: Experiment 3

Simulated malingerers showed significant performance impairments on the BAVLT. However, the abnormal omnibus z-scores of malingering and control subjects showed considerable overlap, so that z-score cutoffs provided limited sensitivity in discriminating simulated malingerers and control subjects with abnormal scores.

We found that two malingering indices that had previously shown sensitivity in distinguishing malingerers from controls, primacy effects and recognition scores, were also helpful in distinguishing participants with abnormal BAVLT scores. Two additional indices, the initial IWI and learning rate, also showed good sensitivity and specificity. Taken together, these four indices provided 80% sensitivity and 80% specificity in identifying abnormal performance due to malingering.

### Limitations

These results should be considered preliminary for several reasons. First, the simulated malingerers in Experiment 3 were experienced with the BAVLT due to the repeated testing in Experiment 2. Second, because of prior exposure to the list used in testing, baseline scores in full-effort conditions would have exceeded their initial baseline scores. Thus, participants in Experiment 3 would need to reduce their acquisition and recall scores considerably before their scores fell into the abnormal range. Thus, learning effects may have reduced the magnitude of malingering-related reductions in comparison with previous studies. For example, Demakis (1999) found that acquisition scores were reduced by 35%, IR scores by 53%, and DR scores by 57% in simulated malingerers, and Sweet et al. (2000) found reductions of similar magnitude. We found reductions of 28%, 40%, and 37% when mean malingering performance was compared to Experiment 2a results, and reductions of only 12%, 27%, and 23% when malingering performance was compared to the results of Experiment 1. These considerations suggest that z-score cutoffs might show higher sensitivity in naïve malingering subjects.

Consistent with previous studies, we found that recognition scores (Bernard et al., 1993; Suhr, 2002; Sullivan et al., 2002) and primacy/recency ratios (Bernard et al., 1993; Suhr, 2002; Sullivan et al., 2002) showed good sensitivity and specificity in distinguishing malingering and control subjects with abnormal performance. Two new metrics, IWIs and learning rate, also showed good metric properties. However, further testing will be needed to determine if these indices are sensitive to simulated malingering in naïve malingering subjects and patients suspected of malingering in clinical populations.

## Introduction: Experiment 4

Previous studies of patients with TBI suggest that the magnitude of deficits on verbal learning depends on injury severity. Patients with moderate-severe TBI typically show significant and long lasting deficits. For example, Draper and Ponsford (2008) examined 60 patients with moderate and severe TBI and found that performance on RAVLT acquisition trials was significantly reduced (Cohen's d = 0.67), with greater impairments seen in patients with more severe TBI. Stallings et al. (1995) studied 40 patients who had suffered severe TBI with the CVLT and RAVLT and found mean z-scores that ranged from −1.0 to −3.4 depending on the normative sample used for comparison. Sweet et al. (2000) found that moderate-severe TBI patients showed impairments of more than one standard deviation in total acquisition, DR, and recognition trials on the CVLT. Jacobs and Donders (2007) compared CVLT performance of 100 controls and 43 patients with moderate-severe TBI tested 3–5 months post-accident and found small but significant reductions in the sTBI group in acquisition and recognition z-scores (−0.70 and −0.61, respectively). Palacios et al. (2013) examined 26 patients with severe TBI (sTBI) and found deficits exceeding two standard deviations in RAVLT acquisition, recall, and recognition scores that correlated with MRI abnormalities in cortical thickness and fractional anisotropy.

In contrast, patients with uncomplicated mild TBI (mTBI) usually show performance within the normal range when tested more than 6 months after injury. For example, Jacobs and Donders (2007) compared 57 patients with mTBI to 100 controls and found that total-acquisition scores in the mTBI group differed from controls by only 0.06 standard deviations. Similarly, Vanderploeg et al. (2005) compared CVLT performance in 254 military veterans with mTBI and 3057 control participants and found differences of less than 0.05 standard deviations in acquisition, DR, and recognition scores. In contrast, several studies have found impaired verbal learning performance in subgroups of mTBI patients with co-morbid PTSD (Verfaellie et al., 2014) and depression (Sozda et al., 2014), and meta-analyses suggest that PTSD alone is associated with significant verbal memory deficits in many patients (Johnsen and Asbjornsen, 2008; Scott et al., 2015).

In Experiment 4, we tested a small group of TBI patients of mixed severity, most with co-morbid PTSD. Based on the results of previous neuropsychological tests of verbal fluency (Woods et al., 2016a) and digit span (Woods et al., 2011b), we anticipated normal performance in mTBI patients, but performance decrements in the sTBI patients.

## Methods: Experiment 4

### Participants and procedures

The methods were identical to those used in Experiment 1. Twenty-eight Veterans with a history of TBI were recruited from the Veterans Affairs Northern California Health Care System patient population. The patients included 27 males and one female between the ages of 20 and 61 years (mean age = 35.5 years), with an average education of 13.4 years. All patients had suffered head injuries and a transient loss or alteration of consciousness, and had received TBI diagnoses after extensive clinical evaluations and standard neuropsychological testing. All patients were tested at least 1 year post-injury. Additional patient characteristics are provided in Supplementary Table S4. Twenty-four of the patients had suffered one or more combat-related incidents, with a loss of consciousness of less than 30 min, no hospitalization, and no evidence of brain lesions on clinical MRI scans. These patients were categorized as mTBI. The remaining four patients had suffered more severe accidents with hospitalization, coma duration exceeding 8 hours, and post-traumatic amnesia exceeding 72 hours. These patients were categorized as sTBI. All patients were informed that the study was for research purposes only and that the results would not be included in their official medical records. Evidence of posttraumatic stress disorder (PTSD), as reflected in elevated scores (>50) on the Posttraumatic Stress Disorder Checklist (PCL)(Weathers et al., 1993), was evident in the majority of the TBI sample. Control participants took the civilian version of the PCL. A comparison of PCL scores in the mTBI patients (mean = 53.0), sTBI patients (mean = 44.5), and Experiment 1 controls (mean = 32.5) showed a highly significant main effect of Group [*F*_(2, 209)_ = 30.39, *p* < 0.0001, ω^2^ = 0.22].

### Detection and exclusion of patients performing with suboptimal effort

Two additional mTBI patient had been identified as performing with suboptimal effort in previous tests, including tests of simple reaction time (Woods et al., 2015d), choice reaction time (Woods et al., 2015a), trail making (Woods et al., 2015b), and spatial span (Woods et al., 2015c). Both patients produced abnormal omnibus scores on the BAVLT and showed signs of malingering, including grossly abnormal recognition scores (respectively 5 and 6) and reduced primacy effects. Therefore, their data were not included in the analysis or the demographic description of the groups.

### Statistical analysis

ANOVA was used to compare the scores from the aggregate control data (Experiment 1 and Experiment 2a) with the z-scores of patients in the mTBI and sTBI groups.

## Results: Experiment 4

Omnibus scores from the mTBI (red circles) and sTBI (blue and white circles) patients are shown in Figure 2. As seen in Figure 4, mTBI patients as a group did not show significant alterations in omnibus z-scores in comparison with control participants [omnibus z-score = 0.02, *F*_(1, 246)_ = 0.05, NS], nor did they show significant alterations in acquisition z-scores [*F*_(1, 246)_ = 0.00, NS], recall-ratio z-scores [*F*_(1, 246)_ = 0.03, NS], recognition scores [*F*_(1, 246)_ = 1.63, NS], total errors [*F*_(1, 246)_ = 0.21, NS], COIs [*F*_(1, 246)_ = 0.99, NS], IWIs [*F*_(1, 246)_ = 1.54, NS], or OLs [*F*_(1, 246)_ = 2.24, *p* < 0.15].

The IWIs of the mTBI patients are shown in Figure 5 (red line) and can be seen to superimpose those of control participants for successive recall positions. Serial position functions of mTBI patients are shown in Figure 6 (solid red line, bottom). While recall was more variable for mid-list words than that of Experiment 1 participants, primacy and recency effects were virtually identical. Two mTBI patients showed abnormal omnibus z-scores (see Figure 4, top) due in one case to impaired acquisition and in the other to borderline impairments in both acquisition and recall-ratio z-scores. Two additional patients showed abnormal recall-ratio z-scores without alterations in acquisition z-scores (Figure 4, bottom).

We also analyzed the relationship between PTSD severity (PCL scores) and test performance. While negative correlations between PCL scores and omnibus z-scores approached significance in the participants in Experiment 1 [*r* = −0.15, t_(193)_ = −2.11, *p* < 0.02, one tailed], they did not reach significance in the smaller mTBI population (*r* = −0.16), nor did PCL correlations with acquisition z-scores (*r* = −0.16) or recall-ratio z-scores (*r* = −0.29) reach significance, although both correlations were in the predicted direction.

Figure 4 includes the z-scores of sTBI patients (blue and white circles). Three sTBI patients performed within the normal range, while one patient showed significant deficits in omnibus z-scores, due primarily to impaired acquisition. The sTBI patient group did not show significant alterations in omnibus z-scores in comparison with control participants [mean = −0.53, *F*_(1, 226)_ = 1.45, NS], although acquisition z-scores showed a weak trend toward reduction [mean = −0.71, *F*_(1, 226)_ = 2.54, *p* < 0.12]. We did not find significant group differences in recall-ratio z-scores [*F*_(1, 226)_ = 0.56, NS], recognition scores [*F*_(1, 196)_ = 1.07, NS], the number of errors [*F*_(1, 226)_ = 0.11, NS], COIs [*F*_(1, 226)_ = 0.51, NS], or OLs [*F*_(1, 226)_ = 1.30, NS]. However, mean IWIs showed a trend toward an increase [*F*_(1, 226)_ = 6.16, *p* < 0.02, ω^2^ = 0.02].

Serial position functions of the sTBI patients are shown in Figure 6 (bottom, dashed red line). Severe TBI patients showed an apparent reduction in primacy and recency effects in comparison with the other participant groups, but further statistical analysis was not undertaken because of the small sample size.

## Discussion: Experiment 4

As in the study of Vanderploeg et al. (2005), we found that military veterans with a history of mTBI showed normal performance on the BAVLT, including total-recall z-scores, total-acquisition z-scores, and recall-ratio z-scores that were virtually identical to those of the control population. Other studies have also found normal performance in most patients with mTBI (Jacobs and Donders, 2007; Whiteside et al., 2015). Four mTBI patients showed abnormalities (more than twice the abnormality rate expected by chance): Two with abnormal omnibus z-scores and two with abnormalities restricted to recall-ratio z-scores. None of these patients showed signs of malingering: All four showed primacy/recency ratios, recognition scores, learning scores, and IWI latencies within the normal range.

We found that increased severity of PTSD symptoms (reflected in elevated PCL scores) tended to be associated with lower BAVLT scores in the control population, and showed non-significant correlations in the predicted direction (Johnsen and Asbjornsen, 2008; Scott et al., 2015) in the mTBI group. However, despite the fact that the mTBI patients had much higher PCL scores than the control participants in Experiment 1, no significant between-group differences were found for any performance metric.

Group abnormalities in acquisition z-scores in the small sTBI group were smaller than those found in most previous studies with one exception (Draper and Ponsford, 2008). However, they failed to reach statistical significance because of the small sample size. There was also a trend toward abnormal IWIs among sTBI patients consistent evidence of slowed processing speed observed in these patients in other studies (Woods et al., 2015a,b).

Abnormalities were most salient in one partially recovered sTBI patient, who showed widespread cortical thinning and frontal lobe abnormalities in quantitative neuroimaging studies (Turken et al., 2009). This patient showed abnormal omnibus and acquisition z-scores, along with two abnormalities characteristic of simulated malingerers: Abnormal recognition scores and abnormal primacy/recency effects. Learning scores were above average, and IWI latencies at recall onset were within normal limits. This patient had shown no signs of malingering on other tests and was considered to be performing with full effort on the BAVLT.

## General discussion

Existing verbal learning tests vary in the time required for testing and the comprehensiveness of scoring. The HVLT is fast, easy to administer, and offers multiple standardized lists for repeated testing. However, it lacks List B and IR trials, has a high incidence of ceiling effects, and shows only moderate test-retest reliability (Woods et al., 2005). The RAVLT is a longer-duration test that includes five List A trials, along with List B, IR, DR, and recognition trials. However, the RAVLT is susceptible to ceiling effects and shows only moderate test-retest reliability (Rezvanfard et al., 2011; Calamia et al., 2013). In addition, depending on the metrics used, the RAVLT can require 10–15 min to score. The CVLT adds semantically cued IR and DR trials, and, unlike the other tests, provides estimates of semantic organization and comprehensive performance measures using scoring software. Ceiling effects appear to be less common on the CVLT than in the HVLT and RAVLT, and test-retest reliability is good for primary measures, but reduced for the supplementary measures (Woods et al., 2006). However, because of the additional cued-recall trials and lengthy “yes/no” recognition test, CVLT administration often requires 30 min. Moreover, CVLT scoring is complex and requires an additional 15 to 25 min to transcribe participant responses so that the computerized scoring program can be used.

In addition, all existing VLTs suffer from a common problem: Inter-examiner differences in scores due to variations in list presentation and scoring (Wiens et al., 1994; Van Den Burg and Kingma, 1999). Inter-examiner effects likely contribute to the differences in the normative data collected in different laboratories. As a result, variations in clinical interpretation can arise based on which normative data set is used for comparison (Stallings et al., 1995).

The BAVLT was designed to minimize inter-examiner and between-laboratory variations in list presentation and scoring. Digitally-recorded words are delivered at calibrated intensities and fixed interword intervals to provide precisely controlled word delivery, and words are presented through headphones to reduce the influence of test-environment acoustics. The examiner also sits outside the subject's field of view to reduce extraneous visual and social cues. Finally, inter-examiner differences in response transcription and scoring are minimized through the use of a simplified one-click scoring system and automated data analysis. As a result, we found no significant inter-examiner differences in scores in the current study.

In comparison with existing tests, the BAVLT is fast and comprehensive: It requires only 10 min to administer and is automatically scored. The BAVLT uses 12-word lists, like the HVLT, but includes all of the trial types used on the RAVLT. Demographic factors, including age, sex, and education, had similar influences on the BAVLT as on the other VLTs. The incidence of ceiling effects on the BAVLT appears to be reduced compared to the HVLT and RAVLT, but slightly higher than on the CVLT. BAVLT test-retest reliability appears to be superior to that of the HVLT and RAVLT, and equal or superior to that of the CVLT. The BAVLT also includes additional measures of semantic organization that are more reliable than those of the CVLT, along with IWI measures that showed high reliability across multiple test sessions.

### Malingering detection

The BAVLT provides malingering detection measures that showed 80% specificity and 80% sensitivity in discriminating individuals with abnormal performance due to malingering from control participants with abnormal performance. The malingering indices also proved useful in Experiment 4, confirming that two mTBI patients who had been identified as performing with poor effort on previous neuropsychological tests appeared to be malingering on the BAVLT.

However, the abnormal sTBI patient in Experiment 4 also showed two indices consistent with malingering (poor recognition and increased IWIs). This suggests that the false positive rate of the malingering indices would likely increase among more markedly impaired patients. For example, patients with Alzheimer's disease would be expected to show abnormal primacy effects (Egli et al., 2014), as well as abnormal recognition (Shapiro et al., 1999; Crane et al., 2012) and learning rate scores. Hence, the utility of the malingering measures would appear to be greatest in identifying malingerers among subjects who would be expected to have normal or near-normal performance.

## Conclusion

The BAVLT is a 10-min computerized verbal learning test that controls for inter-examiner differences in test administration and scoring to enable more valid comparisons across laboratories and normative data sets. The BAVLT shows test-retest reliability that is equal or superior to that of other tests, and provides malingering detection indices to identify individuals who are performing with suboptimal effort. However, further studies with larger patient populations are needed to establish BAVLT sensitivity to clinical deficits that follow TBI and other neuropsychiatric disorders.

## Author contributions

DW conceived the experiments, analyzed the data, and wrote the manuscript. JW assisted with data collection and manuscript preparation. TH assisted with data analysis and manuscript production. EY assisted in programming the experiment, oversaw data collection, and helped to analyze the data and write the manuscript.

## Funding

This research was supported by a VA Research and Development Grants grant CX000583 and CX001000 to DW. The content is solely the responsibility of the authors and does not necessarily represent the official views of Department of Veterans Affairs or the U.S. Government.

### Conflict of interest statement

DW and JW are affiliated with NeuroBehavioral Systems, Inc., the developers of Presentation software that was used to create these experiments. The other authors declare that the research was conducted in the absence of any commercial or financial relationships that could be construed as a potential conflict of interest.
