# Supplementary material for: The Bay Area Verbal Learning Test (BAVLT): Normative Data and the Effects of Repeated Testing, Simulated Malingering, and Traumatic Brain Injury
Source: Front Hum Neurosci. 2017 Jan 12;10:654. doi: 10.3389/fnhum.2016.00654 (PMC5226952; doi:10.3389/fnhum.2016.00654)
Supplement: Supplementary file 1 [file DataSheet1.docx]

|  |  | A1 | A2 | A3 | B | IR | DR |
| --- | --- | --- | --- | --- | --- | --- | --- |
| Exp. 1 | OL | 4.56 (2.26) | 4.16(1.95) | 4.36(2.28) | 4.48(2.19) | 7.35(6.82) | 6.10(7.15) |
|  | IWI | 2.83(4.03) | 2.38(1.46) | 2.27(1.15) | 2.28(1.44) | 3.37(2.07) | 3.63(2.45) |
| Exp. 2a | OL | 3.08(0.98) | 3.23(0.82) | 2.92(0.97) | 3.23(1.24) | 3.45(1.93) | 2.46(1.99) |
|  | IWI | 2.68(1.34) | 2.42(1.30) | 2.16(1.03) | 2.22(1.20) | 3.26(2.21) | 2.96(1.72) |
| Exp. 2B | OL | 3.67(1.56) | 3.46(1.28) | 2.73(0.78) | 3.26(1.24) | 4.09(2.80) | 3.12(2.73) |
|  | IWI | 2.17(1.03) | 2.08(0.87) | 2.02(1.07) | 2.63(1.32) | 2.62(1.32) | 2.61(1.69) |
| Exp. 2c | OL | 3.87(3.72) | 3.21(1.23) | 3.10(2.12) | 3.15(1.07) | 4.25(3.20) | 3.16(2.51) |
|  | IWI | 2.43(1.18) | 2.09(1.01) | 1.96(1.00) | 2.34(1.42) | 2.80(1.26) | 2.98(2.84) |
| Exp. 3 | OL | 3.65(1.36) | 3.96(1.74) | 3.92(2.16) | 3.74(0.97) | 6.23(6.73) | 3.84(2.41) |
|  | IWI | 3.31(1.50) | 3.49(2.47) | 3.69(2.05) | 3.59(1.67) | 4.32(2.76) | 4.30(1.85) |
| mTBI | OL | 3.96(2.04) | 3.30(1.09) | 3.72(1.47) | 4.65(1.95) | 5.25(2.65) | 4.66(4.52) |
|  | IWI | 2.40(0.83) | 2.77(1.19) | 2.57(1.29) | 2.95(1.60) | 3.18(1.67) | 3.38(2.26) |
| sTBI | OL | 4.69(1.85) | 5.06(3.74) | 3.87(1.93) | 3.70(1.39) | 10.64(12.31) | 3.39(1.97) |
|  | IWI | 3.22(2.88) | 2.48(2.04) | 4.90(3.92) | 4.65(1.62) | 5.91(3.19) | 4.70(0.83) |
| **Table S1. Mean onset latencies (OLs) and inter-word intervals (IWIs) during list recall.** Standard deviations are shown in parentheses. See Table 3 for abbreviations. | | | | | | | |

|  | **2** | **3** | **4** | **5** | **6** | **7** | **8** |
| --- | --- | --- | --- | --- | --- | --- | --- |
| **Mean** | **1.20** | **1.42** | **1.47** | **2.07** | **3.17** | **3.76** | **6.37** |
| **SD** | **0.95** | **1.21** | **1.08** | **2.70** | **4.71** | **4.86** | **6.69** |
| **Err %** | **3%** | **3%** | **3%** | **8%** | **12%** | **16%** | **32%** |
| **Table S2. IWIs from the subset of 157 trials with eight responses in Experiment 1.** The data are from 157 trials of all types. SD = standard deviation. Err% = error percentage. | | | | | | | |

|  | **A1** | **A2** | **A3** | **B** | **IR** | **DR** | **Mean** |
| --- | --- | --- | --- | --- | --- | --- | --- |
| **Exp. 1** | **0.28** *(0.24)* | **0.26** *(0.24)* | **0.25** *(0.22)* | **0.25** *(0.22)* | **0.25** *(0.22)* | **0.25** *(0.22)* | **0.25** *(0.12)* |
| **Exp. 2a** | **0.24** *(0.24)* | **0.28** *(0.20)* | **0.31** *(0.24)* | **0.35** *(0.25)* | **0.36** *(0.25)* | **0.38** *(0.26)* | **0.32** *(0.18)* |
| **Exp. 2b** | **0.33** *(0.28)* | **0.37** *(0.30)* | **0.42** *(0.35)* | **0.53** *(0.36)* | **0.41** *(0.32)* | **0.49** *(0.35)* | **0.43** *(0.25)* |
| **Exp. 2c** | **0.35** *(0.28)* | **0.46** *(0.34)* | **0.47** *(0.37)* | **0.34** *(0.27)* | **0.46** *(0.32)* | **0.52** *(0.32)* | **0.43** *(0.16)* |
| **Exp. 3** | **0.23** *(0.20)* | **0.23** *(0.16)* | **0.17** *(0.21)* | **0.26** *(0.29)* | **0.27** *(0.26)* | **0.21** *(0.22)* | **0.23** *(0.16)* |
| **mTBI** | **0.22** *(0.17)* | **0.23** *(0.22)* | **0.20** *(0.21)* | **0.25** *(0.16)* | **0.28** *(0.29)* | **0.24** *(0.28)* | **0.24** *(0.14)* |
| **sTBI** | **0.05** *(0.10)* | **0.26** *(0.21)* | **0.36** *(0.10)* | **0.25** *(0.17)* | **0.14** *(0.09)* | **0.22** *(0.25)* | **0.21** *(0.10)* |
| **TABLE S3. Category organization indices (COIs) for the different experiments.** Values ranged from 0.00 (no category organization) to 1.00 (responses perfectly ordered by category). Standard deviations are shown in parentheses. | | | | | | | |

| Patient | Age | Edu | Severity | Omni Z | Acq Z | Rratio Z | PCL |
| --- | --- | --- | --- | --- | --- | --- | --- |
| Pat01 | 24 | 12 | mTBI | 1.06 | 0.52 | 3.59 | 54 |
| Pat02 | 28 | 12 | mTBI | 0.62 | 0.40 | 2.38 | 66 |
| Pat03 | 31 | 13 | mTBI | 2.48 | 2.39 | 1.66 | 56 |
| Pat04 | 52 | 14 | mTBI | 0.06 | -0.38 | 1.79 | 28 |
| Pat05 | 41 | 14 | mTBI | 1.39 | 0.92 | 3.17 | 45 |
| Pat06 | 20 | 14 | mTBI | -0.35 | -0.32 | 1.80 | 41 |
| Pat07 | 25 | 15 | mTBI | -2.55 | -3.06 | 1.97 |  |
| Pat08 | 28 | 13 | mTBI | -1.27 | -0.88 | -0.15 | 47 |
| Pat09 | 25 | 12 | mTBI | -0.43 | -0.36 | 0.55 | 57 |
| Pat10 | 29 | 12 | mTBI | 0.98 | 1.57 | -0.09 | 54 |
| Pat11 | 47 | 14 | mTBI | 0.14 | 0.40 | 1.31 | 52 |
| Pat12 | 28 | 14 | mTBI | 0.94 | 1.03 | 2.12 | 43 |
| Pat13 | 29 | 13 | mTBI | 1.67 | 1.88 | 3.32 | 27 |
| Pat14 | 61 | 18 | mTBI | -0.26 | -0.03 | 1.38 | 52 |
| Pat15 | 27 | 15 | mTBI | 1.07 | 0.63 | 2.50 | 72 |
| Pat16 | 48 | 13 | mTBI | 0.66 | 0.34 | 3.30 | 59 |
| Pat17 | 49 | 12 | mTBI | -0.33 | -0.17 | 1.18 | 47 |
| Pat18 | 28 | 14 | mTBI | 0.83 | 1.03 | 1.29 | 68 |
| Pat19 | 39 | 13 | mTBI | -0.98 | -0.36 | -1.83 | 64 |
| Pat20 | 25 | 12 | mTBI | -1.18 | -0.81 | -0.77 | 72 |
| Pat21 | 45 | 14 | mTBI | -0.45 | -0.34 | 1.73 | 60 |
| Pat22 | 23 | 14 | mTBI | -1.90 | -1.61 | -1.14 | 67 |
| Pat23 | 29 | 14 | mTBI | -0.76 | -0.54 | 2.80 | 41 |
| Pat24 | 28 | 12 | mTBI | -0.89 | -0.05 | -2.51 | 46 |
| Pat25 | 35 | 12 | sTBI | -1.78 | -2.14 | 1.15 | 59 |
| Pat26 | 46 | 12 | sTBI | 0.13 | 0.20 | 1.42 | 42 |
| Pat27 | 57 | 14 | sTBI | -0.13 | -0.25 | 1.17 | 27 |
| Pat28 | 46 | 14 | sTBI | -0.34 | -0.65 | 3.34 | 50 |
| **Table S4. Patient characteristics.** Age, education, and TBI severity are shown along with Omnibus, Acquisition, and Recall-ratio z-scores. PCL scores on the Post-Traumatic Stress Disorder (PCL) questionnaire are shown. | | | | | | | |

**Appendix A**

**Exps. 1, 2a, 3 and 4.**

**List A:** Raven, Closet, Skiing, Bicycle, Baseball, Sparrow, Deck, Truck, Owl, Boat, Bathroom, Tennis. **Categories:** Birds, Areas of a house, Sports, and Means of Transportation.

**List B:** Tomato, Attic, Pliers, Motorcycle, Wrench, Cabbage, Basement, Taxi, Squash, Van, Garage, Hammer. **Categories:** Vegetables, Areas of a house, Tools, and Means of Travel.

**Experiment 2b.**

**List A:** Binder, Socks, Lemonade, Termite, Soda, Pencil, Belt, Grasshopper, Envelope, Moth, Shirt, Beer. **Categories:** Office items, Clothing, Drinks, Insects.

**List B:** Memo, Sweater, Cello, Dentist, Flute, Folder, Boots, Baker, Notebook, Lawyer, Gloves, Violin. **Categories:** Office items, Office Items, Clothing, Musical Instruments, Occupations

**Experiment 2c.**

**List A:** Squirrel, Dresser, Finger, Poppy, Shoulder, Tiger, Cradle, Orchid, Sheep, Lilac, Sofa, Nose. **Categories:** Animals, Furniture, Parts of the body, Plants

**List B:** Rabbit, Peaches, Elbow, Toothpaste, Leg, Elephant, Plums, Shampoo, Pig, Comb, Strawberries, Neck. **Categories:** Animals, Fruits, Parts of the body, Grooming items
